# Towards the portability of knowledge in reinforcement learning-based systems for automatic drone navigation

**DOI:** 10.7717/peerj-cs.1402

**Published:** 2023-05-19

**Authors:** José M. Barreiro, Juan A. Lara, Daniel Manrique, Peter Smith

**Affiliations:** 1 Departamento de Inteligencia Artificial, Universidad Politécnica de Madrid, Madrid, Spain; 2 Department of Computer Science and Numerical Analysis, Universidad de Córdoba, Córdoba, Spain; 3 University of Sunderland, Sunderland, United Kingdom

**Keywords:** Cyber-physical systems, Reinforcement learning, Knowledge portability, Drones

## Abstract

In the field of artificial intelligence (AI) one of the main challenges today is to make the knowledge acquired when performing a certain task in a given scenario applicable to similar yet different tasks to be performed with a certain degree of precision in other environments. This idea of knowledge portability is of great use in Cyber-Physical Systems (CPS) that face important challenges in terms of reliability and autonomy. This article presents a CPS where unmanned vehicles (drones) are equipped with a reinforcement learning system so they may automatically learn to perform various navigation tasks in environments with physical obstacles. The implemented system is capable of isolating the agents’ knowledge and transferring it to other agents that do not have prior knowledge of their environment so they may successfully navigate environments with obstacles. A complete study has been performed to ascertain the degree to which the knowledge obtained by an agent in a scenario may be successfully transferred to other agents in order to perform tasks in other scenarios without prior knowledge of the same, obtaining positive results in terms of the success rate and learning time required to complete the task set in each case. In particular, those two indicators showed better results (higher success rate and lower learning time) with our proposal compared to the baseline in 47 out of the 60 tests conducted (78.3%).

## Introduction

CPS are complex systems that integrate computational and physical components to perform a given task in the real world. From this definition, it is clear that CPS consist of two well-differentiated but fully inter-related levels. On one hand, the physical elements that are in direct contact with the environment such as sensors or actuators. On the other hand, we have at the computational level we have software elements (such as intelligent agents) in charge of different tasks such as managing user queries, management of incidents arising from the presence of uncertainty, real-time control, knowledge management, and more importantly, uncertainty handling, with several examples of uncertainty drivers given in Asmat, Khan & Hussain (2022).

CPS constitutes a disruptive technology of great importance today (Serpanos, 2018), as it allows us to perform advanced automation and control tasks (Zanero, 2017). It may be applied in different areas ranging from agriculture, manufacturing, critical infrastructure, personalized healthcare, energy management, aircraft controls, to defence systems, and therefore offers important research opportunities in all of them (Liu et al., 2017). Although CPS have important advantages (automation, ease of technology integration, *etc.*), they also pose significant challenges, such as security, reliability, dependability, need for scalability, modularity and composability (Serpanos, 2018); or the need for autonomy without a human in the control loop (Zanero, 2017).

Currently, there are various types of CPS. Of these, one that has sparked great interest recently are the so-called Unmanned Aerial Vehicles (UAV) or drones (Um, 2019), which are aircraft without a pilot on board, and which may be controlled remotely by a pilot or even travel autonomously by means of computational elements. They are of great use in areas such as agriculture, mining, surveillance, military purposes, or medicine, to mention a few (Kumar et al., 2021; Shahmoradi et al., 2020; Mogili & Deepak, 2020).

Generally, most recent research in CPS has focused on the issue of security (Kholidy, 2021; Li & Zhao, 2021). Nevertheless, to the knowledge of the authors, there is barely any work on another essential aspect of CPS, *i.e.,* the importance of the learning of computational elements (for example, intelligent agents) and the positive impact it may have on overcoming the aforementioned challenges of dependability, reliability and autonomy.

In this regard, the use of machine learning techniques appears to be a promising line of research. Especially in environments that require a high degree of autonomy of CPS elements, the use of reinforcement learning (RL) techniques opens up a line of research that we explore in this article. RL is an area of artificial intelligence (AI) that studies how systems size up an environment in an attempt to maximize the notion of reward, and it is based on the concept of reinforcement (positive or negative) (Álvarez de Toledo et al., 2017). There are different approaches to RL which include Q-learning, Monte Carlo methods or, more recently, Deep RL which combines the foundations of RL with the Deep Learning characteristic of artificial neural networks (Botvinick et al., 2020; Wang, Chou & Chung, 2021; Nikita et al., 2021).

In CPS, the knowledge acquired by computational elements by means of approaches such as RL may be highly important if, above all, this knowledge obtained by some of these elements may be exported and shared by others that do not yet possess it. In this regard, the use of knowledge portability approaches is of great interest. The idea behind this approach is that the knowledge acquired when solving a particular task may be used to perform another different (although related) task with a certain degree of success. Recently, this idea has been successfully used in areas such as Education (López-Zambrano, Lara & Romero, 2020), although other applications, such as UAV, can also benefit from this approach.

This article presents a simulated prototype of RL CPS based on intelligent agents that aid in drone automatic navigation and collision avoidance. These systems have been equipped with mechanisms that isolate the knowledge obtained by an agent during their learning process and transfer it efficiently and immediately to other agents without prior knowledge of the environment. This CPS is an improvement adapted for drones, based on an aircraft navigation system previously proposed by the authors (Álvarez de Toledo et al., 2017). Specifically, the contributions of this new work are:

• It proposes a mechanism that lets us isolate the knowledge obtained by an agent during its learning and to separate it from the rest of the tasks (perception, action, etc.), which was not possible in the earlier version of the system and reduced its capacity for knowledge portability.
• It presents a complete and exhaustive study of knowledge portability between agents in different scenarios, which provides an idea of how portable is the knowledge obtained by means of RL techniques.

Therefore, this research article seeks to answer the following research question: To what degree can the knowledge acquired by an agent (drone) in a certain environment be used efficiently by other agent(s) in other different environments?

In the next section we discuss work related to our research. After that, we discuss our system. Then, our experimental design is presented and the results obtained are discussed. The final section of this paper includes the conclusions derived from our work.

## Related Works

The term reinforcement learning (RL) refers to a type of automated learning where the agents that interact in an environment attempt to maximise the concept of reward, so that actions that lead to the achievement of a goal are assessed positively (reward) and those that take them away from the goal are assessed negatively (punishment). They are distinguished from supervised learning approaches as it is not necessary to label the input–output pairs as is the case, for example, in neural networks.

There are multiple RL approaches. Monte Carlo methods are non-deterministic approaches used to simulate complex problems that are difficult to evaluate. They require certain prior experience for learning (Rubinstein, 1981; Kalos & Whitlock, 1991; Ulam, 1991). The Temporal Difference methods make successive predictions of the same value over time and perform what is known as bootstrapping (Sutton, 1978a; Sutton, 1978b; Barto, Sutton & Anderson, 1983). This is an approach where learning is incremental, without the need to wait until the end of a learning episode. On the other hand, the methods called Q-learning use a value-action function to predict the reward that is provided by a specific action in a concrete situation (Watkins & Dayan, 1992). Recently, the fusion of deep learning and RL techniques has proved to be promising. For example, in Botvinick et al. (2020), they provide a high-level introduction to deep RL, discuss some of its initial applications to neuroscience, and survey its wider implications for research on brain and behaviour, concluding with a list of opportunities for next-stage research; in Wang, Chou & Chung (2021), they propose a deep reinforcement learning (DRL) approach to explore better task mappings by utilizing the performance prediction and runtime communication behaviours provided from a simulator to learn an efficient task mapping algorithm; and before in Nikita et al. (2021) they propose a novel approach based on reinforcement learning (RL), wherein a maximization problem is formulated for cation exchange chromatography (biopharmaceutical industry) for separation of charge variants by optimization of the process flowrate.

Given its special connection with this research, it is important to highlight the work of Álvarez de Toledo et al. (2017), where they propose a general RL model independent of input and output types and based on general bioinspired principles that help to speed up the learning process. That model was applied in the air navigation domain, a field with strong safety restrictions, where the perception sensors were based on Automatic Dependent Surveillance-Broadcast (ADS-B) technology. It is a model that uses principles similar to Q-learning and which will be explained later, as the system proposed by the authors in this paper is a drone-related evolution and enhancement of the aforementioned work.

Specifically, RL is now used successfully in the drone industry (Bogyrbayeva et al., 2023). This is mentioned not just in this article, but also in other recently published works. In Faraci et al. (2020), RL has been adopted in the system controller to optimally manage the fleet usage considering the variability of both the bandwidth demand and the green power availability. Hodge, Hawkins & Alexander (2020) describe a generic navigation algorithm that uses data from sensors onboard the drone to guide the drone to the site where a problem is occurring in hazardous and safety-critical situations.

Additionally, in RL scenarios it is important that the learning obtained by an agent to perform a certain task may be used by it or by other agents to perform other different tasks. In this regard, it deals with ideas that have already been proposed and as we shall see later in this article, are worth revisiting. This is the case of works such as those by Konidaris & Barto (2006) and Konidaris, Scheidwasser & Barto (2012), who introduced the use of learned shaping rewards in RL tasks, where an agent uses prior experience on a sequence of tasks to learn a portable predictor that estimates intermediate rewards, resulting in accelerated learning in later tasks that are related but distinct; or by Lazaric (2012), who provided a formalization of the general transfer problem, and identified the main settings which had been investigated so far, and reviewed the most important approaches to transfer in RL.

Outside the field of RL, knowledge portability techniques are also being studied in relation to the drone industry, not just in this work but also in other contemporary works. In Kentsch et al. (2020), the authors study and quantify issues related to the use of transfer learning approaches in their own UAV-acquired images in forestry applications. Chen, Liang & Zheng (2020) propose a learning algorithm that enables a quadrotor unmanned aerial vehicle to automatically improve its tracking performance by learning from the tracking errors made by other UAVs with different dynamics.

Finally, there are three works that are especially linked to this article in that they use knowledge portability (or similar) approaches in RL models applied to drones. In Anwar & Raychowdhury (2020), the authors present a transfer learning based approach to reduce on-board computation required to train a deep neural network for autonomous navigation *via* value-based Deep Reinforcement Learning for a target algorithmic performance. In Venturini et al. (2020), the authors propose a distributed RL approach that scales to larger swarms in UAVs without modifications and can easily deal with non-uniform distributions of targets, drawing from past experience to improve its performance. In Yoon et al. (2019) the authors present an algorithm-hardware codesign for camera-based autonomous flight in small drones that performs transfer and online RL.

Nevertheless, and in spite of their connections to our work (they discuss the idea of using previous RL learning in drones), the aforementioned works belong more to the area of Deep RL, where the models obtained are adjusted in advance (tuning) to test their new use in different tasks (Ladosz et al., 2022). In our research however, learning is not adjusted, rather it is directly transferred for use and is enriched with the new experiences that the agent acquires in the new scenario. Additionally, our work performs a detailed study of how learning behaves when it is shifted from one scenario to another.

With all of the above, and to the best of the authors’ knowledge, this is the first work to make an exhaustive study of the degree of portability of the knowledge extracted by agents (that steer drones and that learn automatically with RL techniques) in certain navigation scenarios, when this knowledge obtained is transferred to other agents to perform different tasks in substantially different scenarios.

## System Description

Throughout this section, we shall provide a detailed description of the proposed system, which is an evolution of the previous system used in the field of air navigation with conventional aircraft, which has been adapted to be used with drones and modified in order to isolate the knowledge extracted by the agents so it may be exported for use by other agents in the same or other scenarios.

It is important to clarify that the developed system is an emulator and the exercises performed are simulations. This is a customary practice in critical areas such as navigation and must be performed before implementation in real environments.

### Antecedents

The previous version of the system was based on a bioinspired RL model that was initially designed to be used in different areas. It was specifically implemented for use in aircraft navigation, with the goal that planes should learn to autonomously travel from one place to another, avoiding collisions with possible obstacles (buildings, other aircraft).

For this, the system was supported by Automatic Dependent Surveillance-Broadcast technology (ADS-B) which allowed it to detect the location of different elements in the environment (point of origin and destination, other planes, etc.) typically with the help of Global Positioning System (GPS) satellites. In traditional navigation environments, these elements are usually detected and communicated by air traffic control towers. Nevertheless, this technology has the advantage that the aircrafts themselves can take decisions at any moment when they encounter any other aircraft or obstacle in their path.

Based on the received readings, throughout the learning process the agent continues to learn how to approach the destination point and how to avoid obstacles, all by means of a simulation. To achieve this, the agent decides on the most convenient action (movement) to be taken at each moment, and after a feedback process (positive or negative), the agent is gradually able to establish positive connections between the perceived situations and convenient actions to be taken at that moment. The possible movements to be made are: ADVANCE, STOP (equivalent to reducing speed), ASCEND (change heading), DESCEND (heading), TURN_RIGHT (heading) and TURN_LEFT (heading). Note that at each moment, the objects (points of origin and destination, location of aircrafts, etc.) have an associated position within a three-dimensional space (X,Y, Z) where “X” and “Y” represent the object’s coordinates taking as reference the ground plane (XY) and “Z” represents the height of the object.

This idea was initially implemented for aircraft, without focusing on whether the knowledge extracted by an agent in a specific environment could be used by other agents in other navigation environments.

The described system, although modular and equipped with certain principles for quick learning by the agents, requires some important changes for its adjustment to other areas and for the knowledge obtained in a scenario to be isolated and exported for use by other agents in different scenarios. For an exhaustive description of the previous version of the system, consult (Álvarez de Toledo et al., 2017).

### Adaptation to drones

A limitation of the system implemented for aircraft was that the movements available to the agents was restricted. This is characteristic of air navigation with planes, as large aircraft have physical limitations with regard to the movements they can make. They undoubtedly constitute one of the most useful, rapid and safe means of transportation, but they possess highly fixed dynamics that prevents them from making certain movements that other types of aerial devices may perform. For example, an aircraft cannot make a tight U-turn within a limited amount of space, nor can it turn on itself as drones can.

Consequently, it was necessary to expand the range of actions available in the action subsystem in order to adapt the previous system for use in drones. More specifically, the six actions meant for aircraft were retained and five new actions were added, which are described in Table 1. Note that the agent is, at all times in the position (X,Y,Z) and, after executing the corresponding action (movement), this position changes according to the indications in the second column of Table 1 (XY is the plane parallel to the ground; YZ is the plane perpendicular to the ground aligned on the length of the agent; <>represents the angle; SIN() represents the sine mathematical operation; *π* is the mathematical constant, with the value 3.141592... ; and finally COS() represents the mathematical operation cosine).

**Table 1:** Description of the new actions implemented.

**Action**	**Effect on the drone position (formulae)**
REVERSE	X = X + SIN (<YZ>)*COS (<XY>+*π*) Y = Y + SIN (<YZ>)*SIN (<XY>+*π*) Z = Z + COS (<YZ>)
MOVE_UP	X = X + SIN (<YZ>−*π*/2)*COS (<XY>) Y = Y + SIN (<YZ>−*π*/2)*SIN (<XY>) Z = Z + COS (<YZ>−*π*/2)
MOVE_DOWN	X = X + SIN (<YZ>+*π*/2)*COS (<XY>) Y = Y + SIN (<YZ>+*π*/2)*SIN (<XY>) Z = Z + COS (<YZ>+*π*/2)
MOVE_RIGHT	X = X + SIN (<YZ>)*COS (<XY>−*π*/2) Y = Y + SIN (<YZ>)*SIN (<XY>−*π*/2) Z = Z + COS (<YZ>)
MOVE_LEFT	X = X + SIN (<YZ>)*COS (<XY>+*π*/2) Y = Y + SIN (<YZ>)*SIN (<XY>+*π*/2) Z = Z + COS (<YZ>)

Note that while the actions performed by aircraft (apart from ADVANCE and STOP) were limited to changes in the heading, drones can directly shift in a specific direction without first having to change their heading. Thus, the action subsystem of our proposal lets us execute all the movements typically associated with a drone, which is actually a supergroup of the movements permitted in a traditional aircraft. Also, note that the STOP action was included in the first version of our system and, although it was not needed for aircraft navigation, it was necessary and useful for drone navigation, since it is one of the most used actions of drones during navigation.

**Figure 1:**
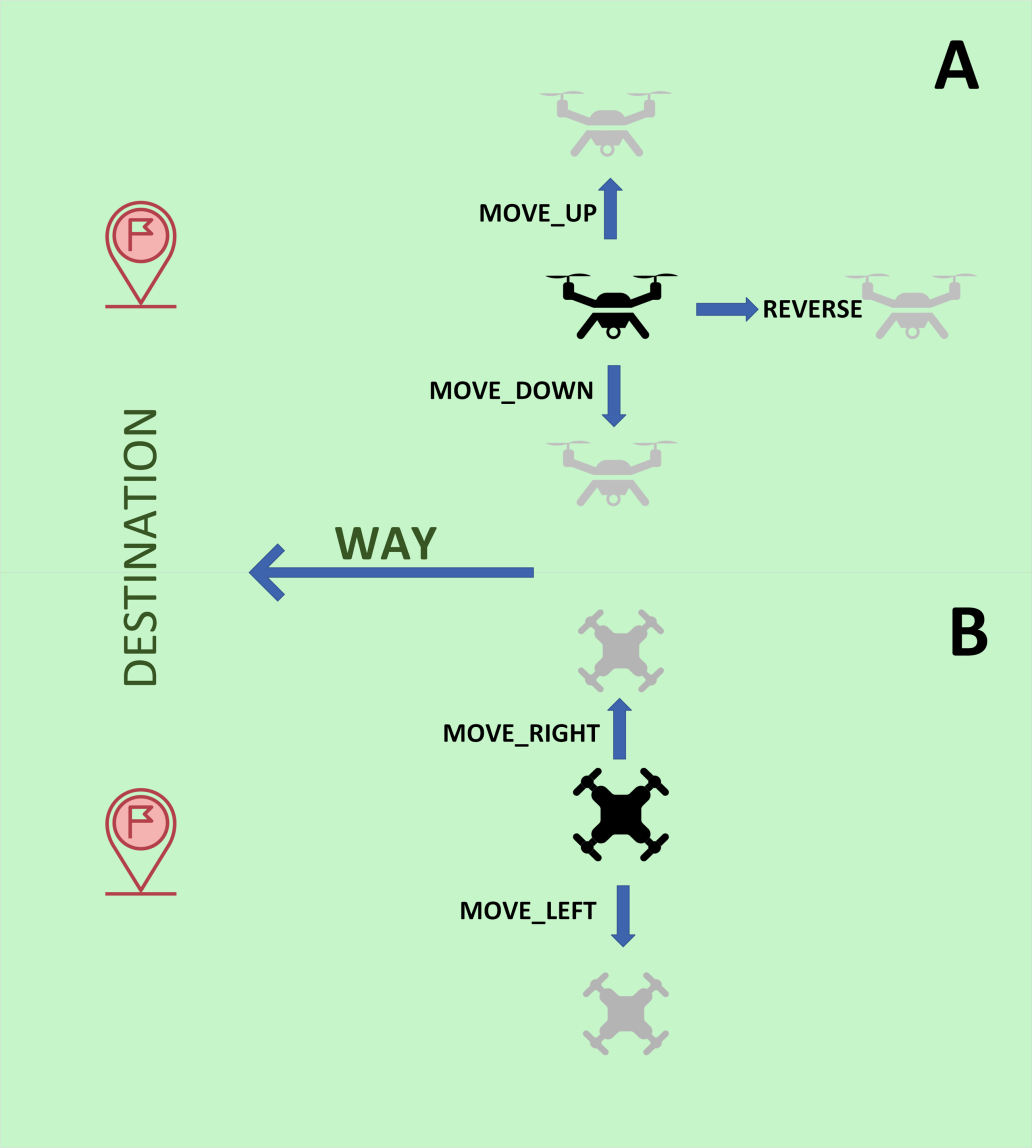
Graphical explanation of the new actions implemented for drones. (A) Side view. (B) Top view.

A graphical explanation of the new actions is presented in Fig. 1, which includes different views of the drone for the sake of clarity.

### Knowledge exportation and importation

In earlier versions of the system for aircraft, the knowledge that the agents progressively acquire over the course of the simulations was distributed over different classes along with other simulation data, which made it difficult to export and then import this knowledge.

In this new version, this knowledge has been redesigned so it may be isolated and separated from the rest of the system data and procedures. In this regard, we have designed two fundamental data structures to manage the knowledge learnt by the agents. On one hand, we have a table which contains the information on patterns perceived in the environment. On the other, we have the information relative to the associations between each perception pattern and the possible actions (movements) to be taken by the drone for said perception pattern.

**Figure 2:**
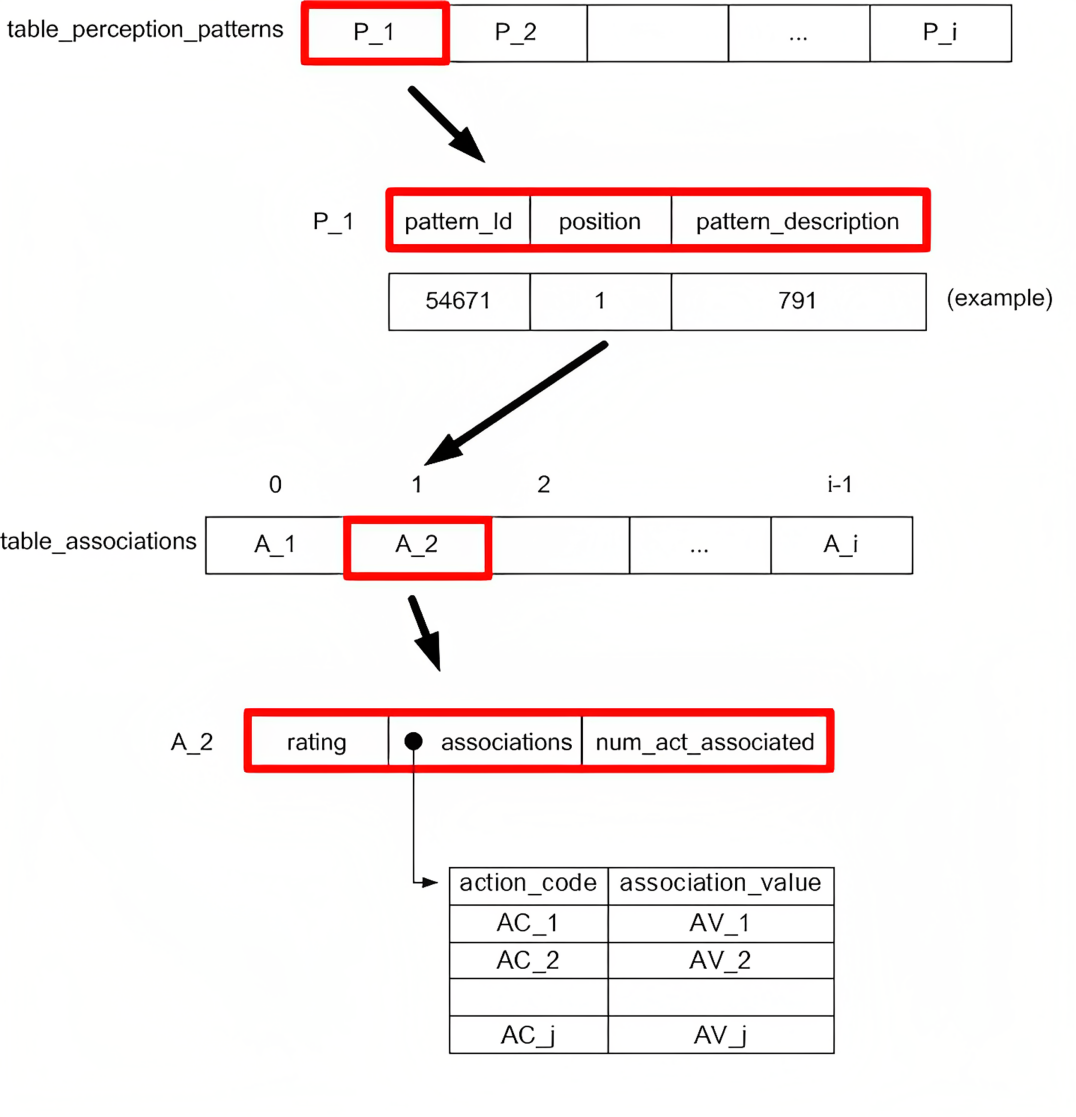
Designed knowledge structures.

These two knowledge structures have been implemented as hash tables, and are described in Fig. 2. In the upper part of the figure, we see how the table on perception patterns (table_perception_patterns) consists of a set of elements that represent each perception pattern(P_1, P_2, …), each containing an identifier (pattern_Id), the pattern position (position) and a coded description of the perception pattern (pattern_description). Note that each pattern has a unique identifier which is generated by the system incrementally as the environment is progressively discovered by the agents. In turn, the position of the perception pattern is used to learn which element from the table of associations corresponds to the specific perception pattern. Finally, the description of each perception pattern is the result of the (coded) concatenation of the information that the perception system receives from the environment in relation to the agent’s position, the detection of obstacles, and the detection of other agents (see more details on the description of the pattern in Álvarez de Toledo et al. (2017)). It is necessary to export this information so that when imported, the agents can already draw upon the knowledge of the environment, marked by the explained patterns and their description.

In the lower section of the figure, we see the table of associations (table_associations). Here, we may interpret a series of numbered positions that are marked from the “position” field in the table on perception patterns. Each position contains a set of elements that are the assessment of the perception pattern, rating (positive or negative and with a greater or lower value depending on their proximity or distance from the destination point and the presence of obstacles—more details on the assessment subsystem in Álvarez de Toledo et al. (2017)); a pointer that indicates another table (associations) that contains the association values of said perception pattern with each of the associated actions and an integer (between 0 and 10) that represents the number of actions associated with each perception pattern (num_act_associated) from the 11 possible actions that may be executed by the drone. The aforementioned table of associations stores sets that represent the degree of association (association_value) of the perception pattern in question with each one of the associated actions (action_code). Note that a perception pattern may not be associated with all possible actions, only those that the agent has experienced until then for each perception pattern during the learning process.

Once this information is isolated, it may be exported for subsequent importation. For this, we have opted to use text files (two: one for the table on perception patterns and another for the table of associations) with a structure similar to that of the explained tables. The knowledge export procedure is responsible for creating and opening these files, dumping the perception patterns into the file in question and finally, dumping the associations in the corresponding file. This procedure is included in Algorithm 1, where the two data structures described, the number of pattern perceptions, and filenames assigned by the user to the files to be generated, are entered and it outputs said files with the exported knowledge.

**Table:** 

ALGORITHM 1
** Input**: table_perception_patterns, table_associations, num_perception_patterns, name_file_patterns, name_file_associations
**Output**: <name_file_patterns>.pdp, <name_file_associations>.aso
**Procedure**:
1. Create file <name_file_patterns>.pdp
2. Create file <name_file_associations>.aso
3. Open file <name_file_patterns>.pdp
4. Open file <name_file_associations>.aso
5. For *i* = 1 …num_patrones_percepcion
// The perception patterns are dumped
5.1. pos = table_perception_patterns[i].position
5.2. Write (<name_file_patterns>.pdp, table_perception_patterns[i].pattern_Id + TAB) //tabulator
5.3. Write (<name_file_patterns>.pdp, pos + TAB)
5.4. Write (<name_file_patterns>.pdp, table_perception_patterns[i].pattern_description + EOL) //end of line
//The associations are dumped
5.5. Write (<name_file_associations>.aso, table_associations[pos].num_act_associated + BLANK)
5.6. Write (<name_file_associations>.aso, table_associations[pos].rating + EOL)
5.7. For *j* = 1 …table_associations[pos].num_act_associated
5.7.1. Write (<name_file_associatios>.aso,
table_associations[pos].associations[j].association_value + BLANK)
5.7.2. Write (<name_file_associations>.aso,
table_associations[pos].associations[j].action_code + EOL)
End For (5.7)
End for (5)
6. Write (<name_file_patterns>.pdp, EOF) //end of file
7. Write (<name_file_associations>.aso, EOF)
8. Close file <name_file_patterns>.pdp
9. Close file <name_file_associations>.aso

**Figure 3:**
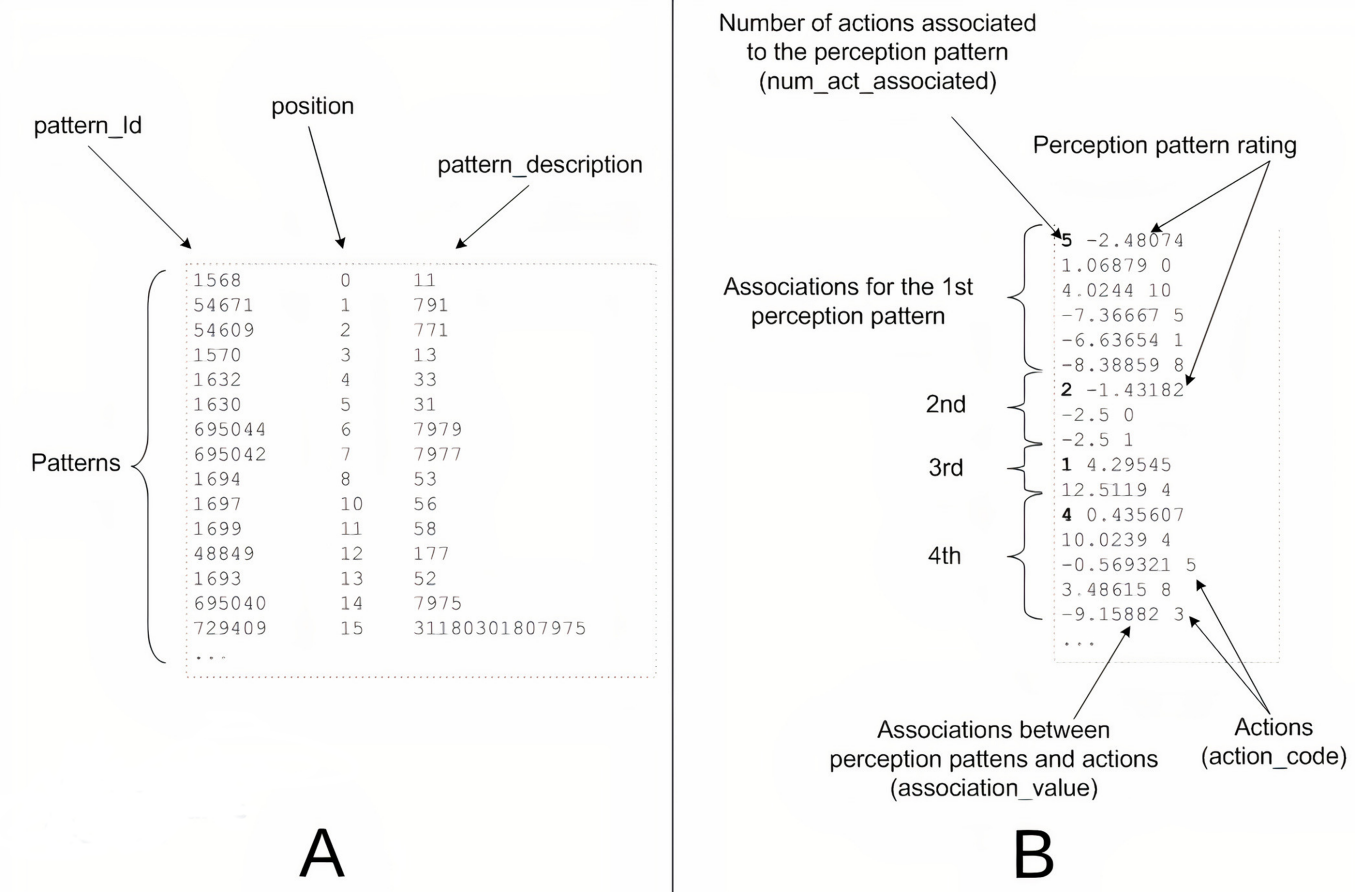
Example of real fragments from exported files. (A) Perception patterns file (.pdp). (B) Association file (.aso).

As an example, Fig. 3 contains a screenshot of a real fragment (within the dotted outline) of a perception pattern file generated by the system (Fig. 3A) and another with a fragment from an associations file (Fig. 3B). The patterns file contains a line for each pattern(identifier, position and description). The associations file is more complex and for each perception pattern, it includes the number of associated actions (highlighted in bold in the figure) and the assessment of the pattern in the first line followed by a number of lines equal to the number of associated actions, including in each line the value of the association with the corresponding action, followed by the code of said action.

The import procedure is similar but in reverse order. That is to say, the data stored in the patterns and associations files are dumped into the perception patterns table and the associations table respectively, and the agent is thus ready to use the imported knowledge. As a procedure analogous to the exporting (but in reverse), we have not provided an added description of the import procedure, for simplicity’s sake.

## Experimental Design

To assess the system presented in the previous section, we have designed a validation strategy that considers different learning scenarios where one (or more) drone(s) must move from a given starting point to a specified destination point, avoiding collisions with defined obstacles and with other drones present in the environment. The different scenarios are detailed in the next subsection.

For each scenario, we have a training procedure with the goal of achieving learning by the agents and its later exportation. Next, this knowledge is imported by other agents without prior knowledge of the environment in question and the utility of this knowledge in other scenarios has been tested. This test was performed according to a series of metrics presented later in this paper.

Note that we have performed a system implementation that allows us to perform simulations. For this, each agent must face the environment in which it must learn and for this, it has a maximum number of movements in which to achieve its destination (called “cycles”). If the destination is achieved without exhausting the maximum number of cycles, the agent is stated to have obtained a “success”. Otherwise, the agent obtains a “failure”. In any case, the agent repeats this procedure in a loop, therefore, in each iteration, it possesses the knowledge obtained in the previous iterations (accumulated knowledge). Each of these iterations is called “attempt”. In each experiment, 15,000 attempts for each simulation have been considered and a maximum of 600 cycles per attempt.

### Description of the learning scenarios

The experiments performed have considered a total of five different scenarios. They have varying numbers and positions of agents, as well as varying quantities and distributions of obstacles in order to have sufficient variety so we may obtain conclusions of interest. The scenarios considered are described in Table 2. Note that we have increased complexity to check the evolution of the performance in learning portability. For instance, we have started with a simple scenario with just one agent and no obstacles, then two agents with no obstacles, and finally one agent with obstacles of different types.

**Table 2:** Explanation of the simulation scenarios posed.

**Id**	**Description**	**Picture**
Sc1	There is no obstacle. A single agent must reach from the starting point (20, 60, 5) to the destination point (20, 0, 0) and land there, only avoiding collision with the ground.	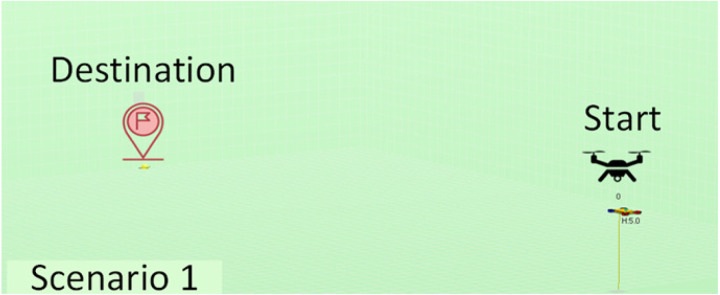
Sc2	There is no obstacle. Two agents located initially at the positions (15, 60, 5) and (25, 60, 5) respectively must reach the destination point (20, 0, 0) and land, avoiding collisions with the ground and with each other.	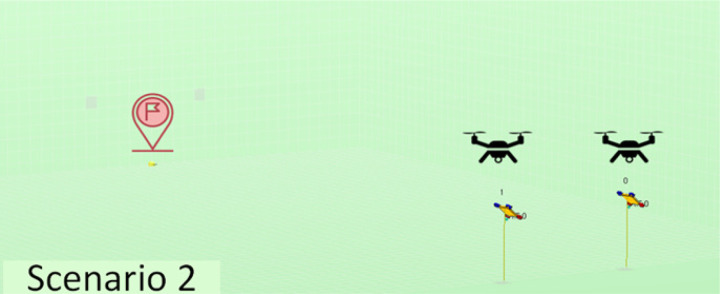
Sc3	A single agent must reach from the starting point (20, 60, 5) to the destination point (20, 0, 0) and land there, avoiding collision with the ground and with three fixed towers of a certain width standing in its way.	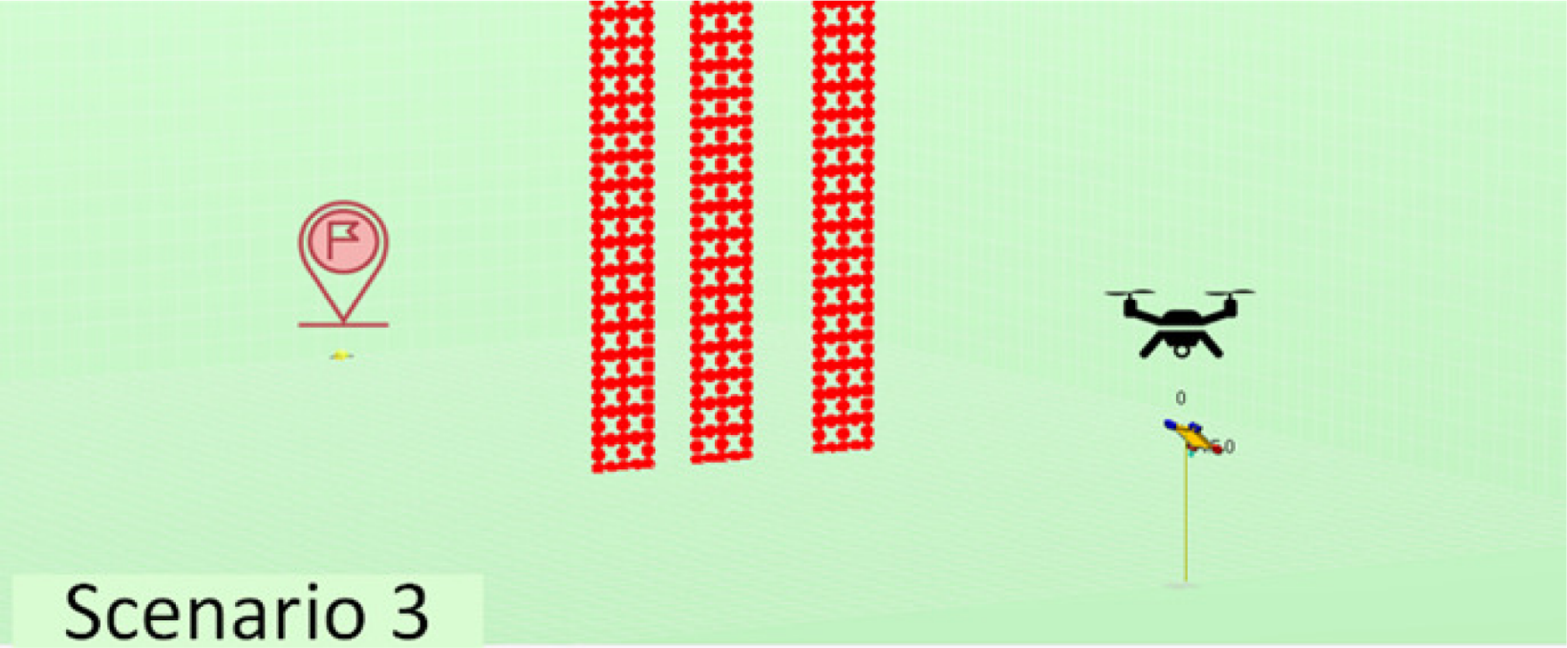
Sc4	A single agent must reach from the starting point (20, 60, 5) to the destination point (20, 0, 0) and land there, avoiding collision with the ground and with twenty fixed narrow towers standing in its way.	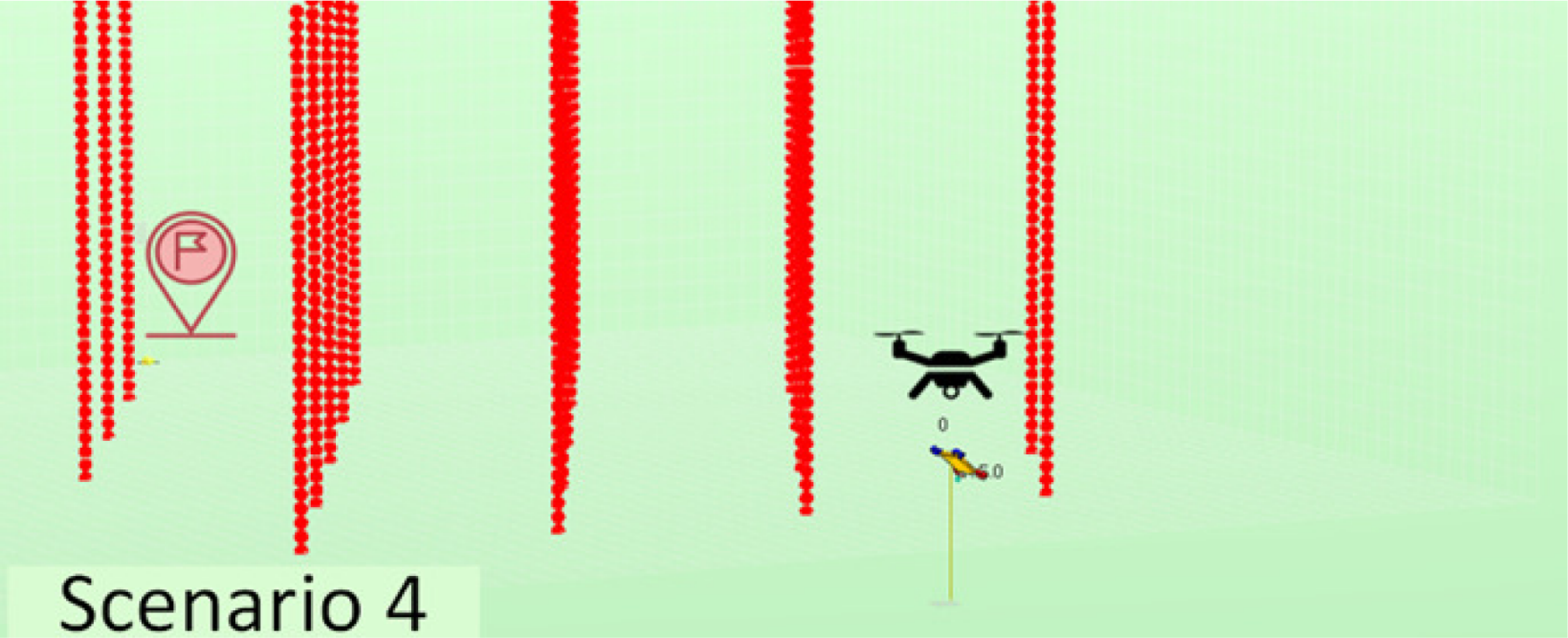
Sc5	A single agent must reach from the starting point (20, 60, 5) to the destination point (20, 0, 0) and land there, avoiding collision with the ground and with a fixed tower of a certain width standing in its way.	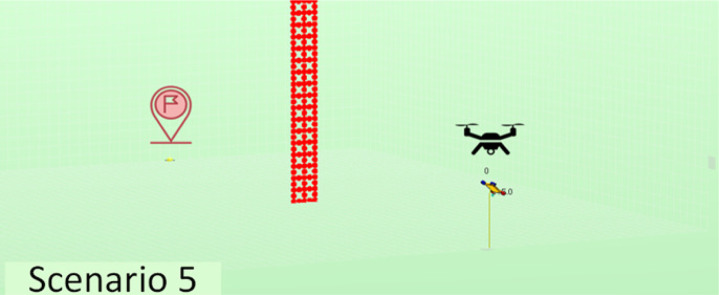

### Metrics used

In order to assess the utility of the learning imported by an agent in an unknown scenario, the following metrics were used:

• Average number of cycles per attempt (Avg_CI): This considers the number of cycles that the agent has invested in each attempt and calculates the average of said values. The lower the value, the quicker the agent is able to perform its task and therefore it implies better learning.
• Success rate (%Success): represents the number of successful attempts in relation to the total number of attempts for each simulation.
• Simulation time (T_Sim): represents the amount of time required by the agent (or agents) to complete the simulation. The lesser the time, the quicker the agent has learnt.

These metrics are especially significant for analysing the results of this article, as they constitute indicators that are directly related to learning efficiency, which is precisely what we seek to study.

## Results

This section displays the results obtained in each scenario when using the knowledge generated in the other scenarios, in an attempt to measure the degree of portability of this knowledge between different scenarios and thus respond to the research question posed in this article.

For this, we shall now describe each scenario mentioned in the previous section, and we shall demonstrate with graphs and tables, the results that have been obtained with reference to portability, using the aforementioned metrics as reference.

### Scenario 1 (Sc1)

The results displayed in Table 3 have been obtained from the tests relative to this scenario. This table format will be used for all the scenarios and this is why it requires a prior explanation. The rows represent the different metrics considered. The final column always displays the results obtained by the agents in the learning process of the scenario (Scenario 1 in the case of Table 3), without knowledge import, therefore this is the baseline column for each scenario. The rest of the columns display the results obtained in the analysed scenario (Scenario 1 in case of Table 3) using the knowledge generated previously in the rest of the scenarios and subsequently imported (the best values for each metric are highlighted in bold in the knowledge import columns, with the exception of the baseline column).

**Table 3:** Results obtained for Scenario 1.

	Sc2 → Sc1	Sc3 → Sc1	Sc4 → Sc1	Sc5 → Sc1	Sc1 (baseline)
Avg_CI	80.8	123.48	106.28	**80.22**	101.56
%Success	**100**	98.67	99.33	**100**	98.33
T_Sim	**3′17″**	3′42″	4′16″	10′31″	4′24″

**Notes.**

The best values for each metric are highlighted in bold. The values that worsen the baseline are underlined.

The analysis of the results of the table shows that with regard to the average number of cycles per attempt, using the knowledge of Scenarios 1 (own), 2 and 5 improves the baseline data for Scenario 1, but worsens when using the knowledge of Scenarios 3 and 4 (the values that worsen the baseline are underlined). The success rate, the primary indicator, improves in all cases with reference to the baseline. The simulation time improves (is reduced) in all cases except when using the knowledge of Scenario 5.

**Figure 4:**
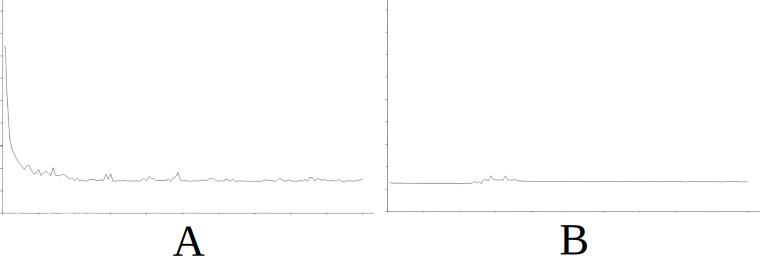
Comparing the evolution of learning (cycles per attempt) of the baseline with the best case of importation for Scenario 1. (A) Baseline. (B) Importing knowledge from Sc5 (best case).

**Figure 5:**
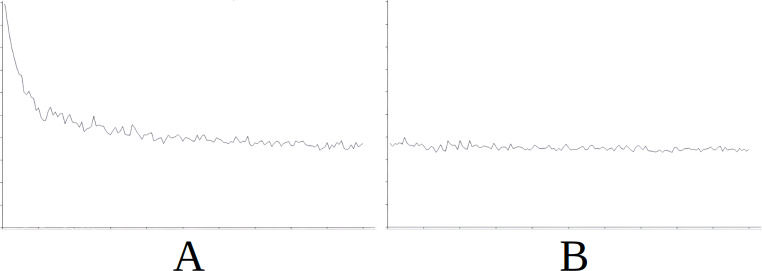
Comparing the evolution of learning (cycles per attempt) of the baseline with the best case of importation for Scenario 2. (A) Baseline. (B) Importing knowledge from Sc1 (best case).

**Figure 6:**
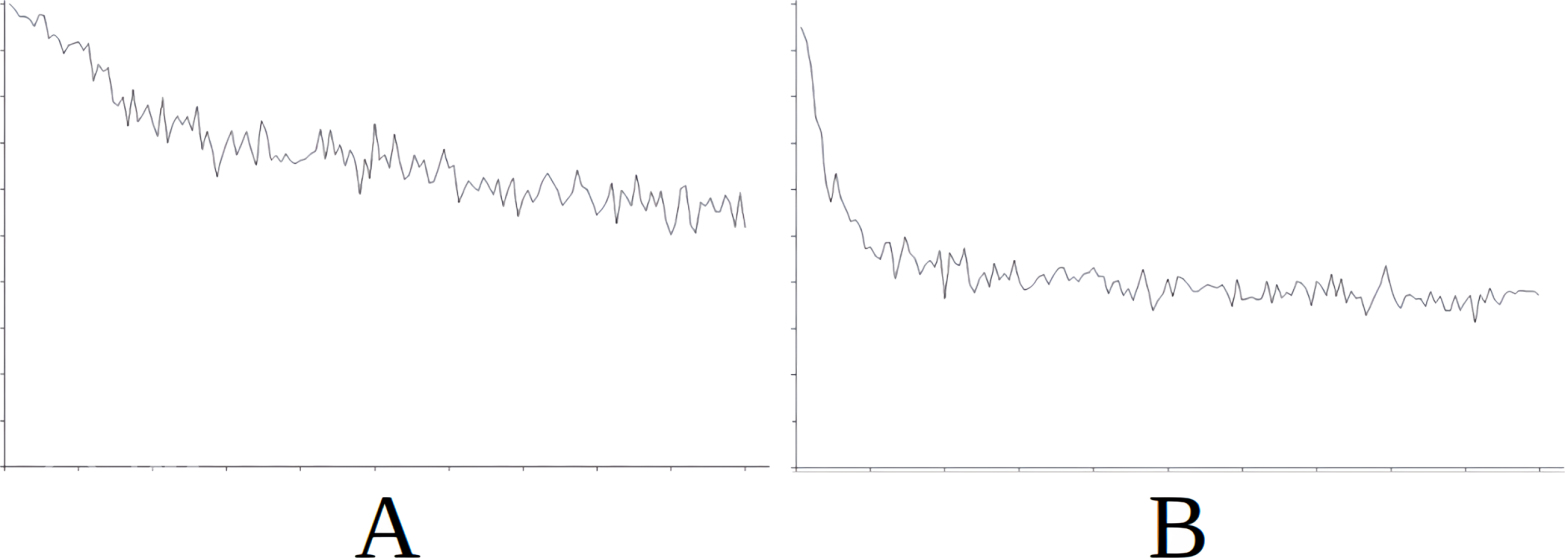
Comparing the evolution of learning (cycles per attempt) of the baseline with the best case of importation for Scenario 3. (A) Baseline. (B) Importing knowledge from Sc2 (best case).

**Figure 7:**
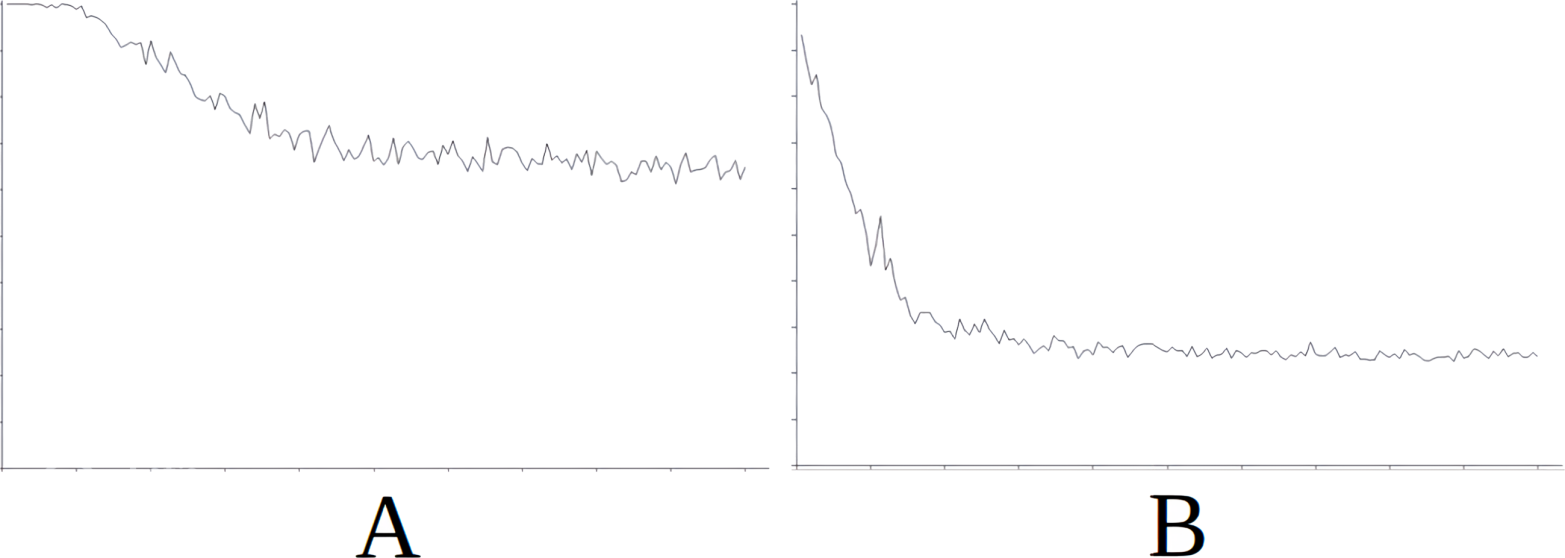
Comparing the evolution of learning (cycles per attempt) of the baseline with the best case of importation for Scenario 4. (A) Baseline. (B) Importing knowledge from Sc5 (best case).

**Figure 8:**
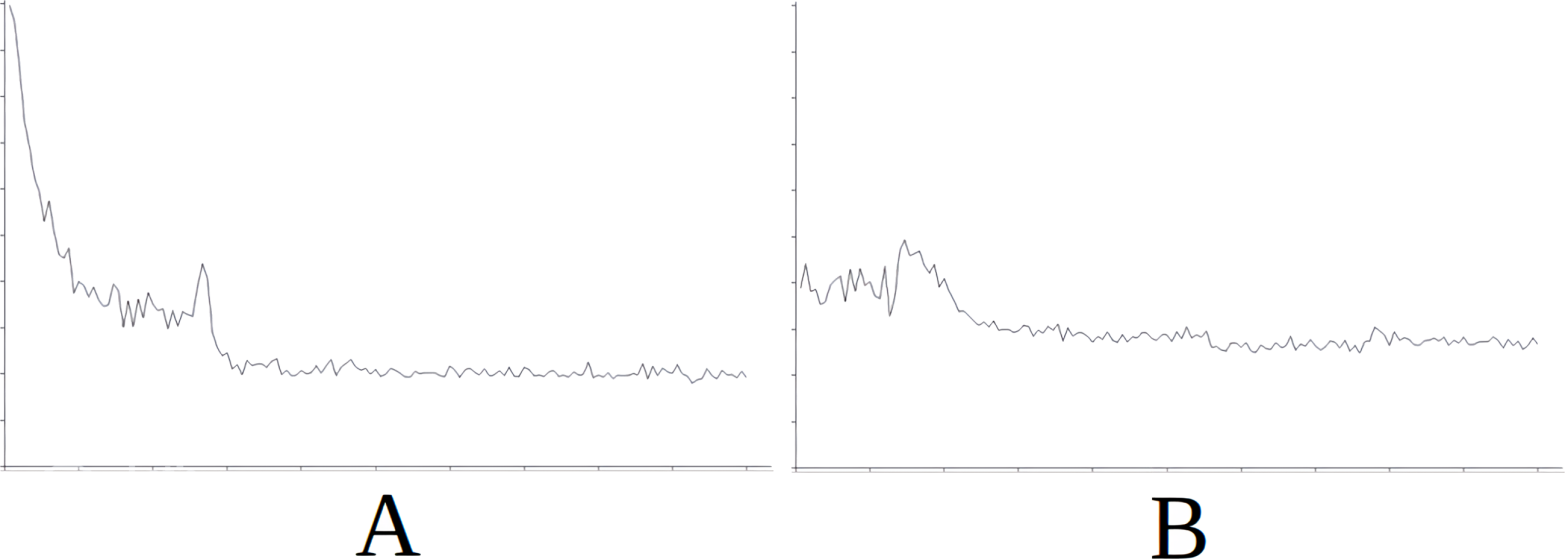
Comparing the evolution of learning (cycles per attempt) of the baseline with the best case of importation for Scenario 5. (A) Baseline. (B) Importing knowledge from Sc3 (best case).

As part of the results for this scenario, Fig. 4 displays the evolution of learning in the baseline (Fig. 4A) and the evolution of learning in the best of the cases with importing knowledge (with reference to the success rate) which is Scenario 5 in this case (Fig. 4B). For the sake of simplicity, this figure does not include information on the axes as it is identical in meaning and scale for all the figures and additionally, what interests us is the evolution of the graph. The horizontal axis simply represents each attempt in the simulation (range [0–15000], with the axis divided into sections of 1,500 attempts each) and the vertical axis represents the number of cycles needed for success in each attempt (range [0–600], with the axis divided into sections of 60 cycles each). The above applies also to Figs. 5, 6, 7 and 8.

In Fig. 4 we see that in the first attempts, the agent has difficulties in attaining the goal, using a high number of cycles, until learning is stabilised around attempt 1,500. Nevertheless, this stability is noted in Fig. 4B from the start of the simulation, therefore the knowledge imported from Scenario 5 is useful from the very beginning.

### Scenario 2 (Sc2)

The results displayed in Table 4 have been obtained from the tests relative to this scenario. In this case, for the three indicators, there are two scenarios where the results improve the baseline, and another two where they do not.

Figure 5 demonstrates the baseline evolution of learning (Fig. 5A) compared to an equivalent simulation importing the knowledge of Scenario 1 in this case (Fig. 5B), which gives the best success rate after import. Again, we see that the imported knowledge is of great use, given that the agent achieves success from the starting cycles of the simulation, and maintains this stable trend (Fig. 5B). Without this imported knowledge the learning process is much longer (Fig. 5A).

### Scenario 3 (Sc3)

The results displayed in Table 5 have been obtained from the tests relative to this scenario. In this case, with the exception of the indicator Avg_CI in the import from Scenario 4 to 3, the imported knowledge improves the baseline results.

Figure 6 demonstrates the baseline evolution of learning (Fig. 6A) compared to an equivalent simulation importing the knowledge of Scenario 2 in this case (Fig. 6B), which gives the best success rate after import. In this case we observe that although the agent still has to learn in the first attempts even when knowledge is imported, learning is stabilized quite rapidly from attempt 3,000 onwards (Fig. 6B). However, without this imported knowledge the learning process is much longer and unstable (Fig. 6A).

**Table 4:** Results obtained for Scenario 2.

	Sc1 → Sc2	Sc3 → Sc2	Sc4 → Sc2	Sc5 → Sc2	Sc2 (baseline)
Avg_CI	**213.96**	312.21	464.08	232.87	253.17
%Success	**99**	91	65	96	93
T_Sim	**6′5″**	7′41″	9′43″	23′32″	7′48″

**Notes.**

The best values for each metric are highlighted in bold. The values that worsen the baseline are underlined.

**Table 5:** Results obtained for Scenario 3.

	Sc1 → Sc3	Sc2 → Sc3	Sc4 → Sc3	Sc5 → Sc3	Sc3 (baseline)
Avg_CI	383.24	261.09	464.01	**235.27**	428.33
%Success	69	**90**	63	87	61
T_Sim	28′31″	17′52″	25′30″	**17′40″**	34′51″

**Notes.**

The best values for each metric are highlighted in bold. The values that worsen the baseline are underlined.

### Scenario 4 (Sc4)

The results displayed in Table 6 have been obtained from the tests relative to this scenario. We can clearly see that the importing of this knowledge improves all indicators in all the imports performed.

**Table 6:** Results obtained for Scenario 4.

	Sc1 → Sc4	Sc2 → Sc4	Sc3 → Sc4	Sc5 → Sc4	Sc4 (baseline)
Avg_CI	351.13	246.81	216.12	**189.12**	441.07
%Success	85	91	92	**95**	67
T_Sim	30′42″	21′15″	18′49″	**16′14″**	39′50″

**Notes.**

The best values for each metric are highlighted in bold. The values that worsen the baseline are underlined.

Figure 7 demonstrates the baseline evolution of learning (Fig. 7A) compared to an equivalent simulation importing the knowledge of Scenario 5 in this case (Fig. 7B), which gives the best success rate after import. In this case, once again, although the agent still needs time to stabilise learning (Fig. 7B) after importing knowledge, the time taken is much less that when knowledge is not imported (Fig. 7A).

### Scenario 5 (Sc5)

The results displayed in Table 7 have been obtained from the tests relative to this scenario. In this case, the number of cycles per attempt does not improve in most cases of importation, but the success rate and the simulation time both improve (with one exception in each case).

**Table 7:** Results obtained for Scenario 5.

	Sc1 → Sc5	Sc2 → Sc5	Sc3 → Sc5	Sc4 → Sc5	Sc5 (baseline)
Avg_CI	191.76	**155.86**	179.33	350.41	171.13
%Success	95	96	**97**	78	94
T_Sim	33′34″	**7′40″**	8′10″	12′16″	28′51″

**Notes.**

The best values for each metric are highlighted in bold. The values that worsen the baseline are underlined.

Figure 8 demonstrates the baseline evolution of learning (Fig. 8A) compared to an equivalent simulation importing the knowledge of Scenario 3 in this case (Fig. 8B), which gives the best success rate after import. In this case, when knowledge is imported, learning is found to be quite stable (with the exception of certain peaks around attempt 2,000) from the beginning (Fig. 8B) when compared to the baseline (Fig. 8A) where the early attempts constitute slow learning and with several failures, until learning is stabilised around attempt 7,500.

## Conclusion

This article proposes a simulated system for drone navigation based on a RL model. This system allows drones to arrive at a specific destination point completely automatically, avoiding physical obstacles and collisions with other drones. It is the evolution of a previous system implemented by the authors for drones with modifications to the range of possible movements by agents in the surroundings (Álvarez de Toledo et al., 2017).

Additionally, the new system has been equipped with new mechanisms that allow the knowledge obtained in a scenario to be separated from the rest of the system data and procedures, so it may be exported for later use in other scenarios. Specifically, this article studies the degree of portability of knowledge between different scenarios. For this, we have performed learning simulations for five significantly different scenarios and the knowledge acquired in each scenario has been transferred to the rest in order to determine its utility in learning.

A total of 20 knowledge transfers were made in all the five scenarios. In each transfer, three different metrics were studied, leading to 60 portability results. Of these 60 results, positive results were obtained in 47 cases in comparison to the baseline scenario (78.33%) thus giving us an affirmative answer to the research question posed in this article.

When analyzing each scenario independently, the best transfer results were displayed in Scenario 4 (when knowledge was transferred from the rest of the scenarios to Scenario 4). In this case, all metrics improved after knowledge was transferred in all cases. The worse results were those of Scenario 2 (when knowledge was transferred from the rest of the scenarios to Scenario 2), with improvement observed in half of the transfers and worse results in the other half. In the remaining scenarios, results were generally positive, but with certain exceptions. This seems to indicate that the presence of small obstacles (Scenario 4) is a pattern that is simple to learn with previous knowledge of other surroundings, even when these do not have obstacles. Nevertheless, the presence of various drones (Scenario 2) generates patterns that are more complex to learn, therefore, prior knowledge with simple fixed obstacles is not of great use.

An analysis of the scenarios in pairs shows a high degree of learning portability between Scenarios 1 and 2, and in both directions. These are scenarios without obstacles, but with different number of drones. On the other hand, there is low portability between Scenarios 1 and 5, even though the only difference between them is the presence of an obstacle of medium size. This appears to indicate that the presence of medium or large obstacles requires an extra knowledge that cannot be imported from other scenarios without obstacles of this type. In general, it appears that the biggest complication lies in achieving a good transfer towards Scenarios 2 (multiple drones) and 5 (medium–large obstacle).

These results may serve as a benchmark in real learning environments without the possibility of simulations and where experimentation may be expensive. Additionally, the procedures conducted in this article may serve as a reference for other similar studies of knowledge portability in the field of artificial intelligence in general, and machine learning in particular. The ideas of portability presented in our paper can be used in any learning problem as they are general enough. However, the implementation details of each particular system will have to be modified and adapted to include our ideas. This would be the main difficulty in generalizing our ideas for use in other domains. The general nature of the model and of the portability procedure are the strong points of this study. Of course, the study was limited to environments with drones where they can interact with each other and with fixed obstacles, and it must be considered as a preliminary analysis in a limited set of scenarios. In addition, our study is only a simulation in a very controlled environment where drones do not have to face external constraints and circumstances, such as weather conditions, limitations in the use of resources or legal issues.

Some lines of research that may be worth examining in the future are the following:

• To expand the study to include a greater number of scenarios, especially scenarios with multiple drones interacting with each other and with large obstacles present in the trajectory of these drones.
• To take a first step towards converting the implemented system(simulation) into reality. For this, real drones are required with processing systems that include the logic of the presented system. Additionally, these drones must be equipped with a perception system(sensors) that allows them to detect obstacles and other drones, and to know their position relative to the destination point (possibly GPS). It must be possible to map the set of movements of the simulated agent on to the movement orders of a drone, probably by means of a movement interface facilitated by its operating system.
• For a more efficient and sustainable learning process of the agents, an option would be to attempt to reduce the number of perception patterns, which would lead to less storage and fewer decisions to analyse. This may be achieved by grouping patterns that are very similar using data mining (clustering) techniques, for example. It must be studied if the reduction in patterns, along with improved sustainability, allows us to maintain a certain level of quality with regard to learning (success rate).

## Additional Information and Declarations

### Data Availability

The following information was supplied regarding data availability: The code is available at GitHub and Zenodo:
https://github.com/in2latoj/MARSADA/tree/Marsada
in2latoj. (2023). in2latoj/MARSADA: Marsada2.0 (Marsada). Zenodo. https://doi.org/10.5281/zenodo.7614681
The data is available at Zenodo: Juan Alfonso Lara. (2023). Data used in Knowledge Transfer Research for Drone Navigation [Data set]. Zenodo. https://doi.org/10.5281/zenodo.7600381
